# Estradiol alters the immune-responsiveness of cervical epithelial cells stimulated with ligands of Toll-like receptors 2 and 4

**DOI:** 10.1371/journal.pone.0173646

**Published:** 2017-03-15

**Authors:** Behnia S. Lashkari, Dilly O. C. Anumba

**Affiliations:** Academic Unit of Reproductive and Developmental Medicine, Department of Human Metabolism, University of Sheffield, Jessop Wing, Sheffield, United Kingdom; University of Kansas Medical Center, UNITED STATES

## Abstract

The mucosa of the female reproductive tract plays a pivotal role in host defence. Pregnancy must alter immunological mechanisms at this interface to protect the conceptus. We sought to determine how estradiol (E_2_) alters the immune-responsiveness of cervical epithelial cells to ligand stimulation of Toll-like receptor (TLR)-2 and -4. Human ectocervical epithelial cells (HECECs) were cultured and co-incubated with two concentrations of E_2_ and peptidoglycan (PGN) or lipopolysaccharide (LPS) over durations that ranged between 10 minutes and 18 hours. Cytometric Bead Array was performed to quantify eight cytokines in the supernatant fluid. In response to PGN, HECECs co-incubated with E_2_ released lesser quantities of IL-1ß and IFNγ, higher levels of RANTES, and variable levels of IL-6 and IL-8 than those not exposed to E_2_. In contrast, HECECs co-incubated with LPS and E_2_ secreted increased levels of IL-1ß, IL-6, IL-8, and IFNγ at 2 and 18 hours than HECECs not exposed to E_2_, and reduced levels of RANTES at same study time-points. Estradiol alters the immune-responsiveness of cultured HECECs to TLR2 and TLR4 ligands in a complex fashion that appears to vary with bacterial ligand, TLR subtype, and duration of exposure. Our observations are consistent with the functional complexity that this mucosal interface requires for its immunological roles.

## 1. Introduction

The epithelium of the female reproductive tract plays a pivotal role in host defence against pathogens. It secrets specific mucosal proteins such as mucins and defensins [1,2], and recognises pathogen-associated molecular patterns (PAMPs) on microbes [3,4] through pattern recognition receptors (PRR) of the Toll-like receptor (TLR) family amongst others [5,6]. The epithelium also provides a mechanical barrier against microbes, and secretes cytokines and antimicrobial peptides which coordinate the local innate and adaptive immune responses [7,8]. There is emerging evidence that these innate immunological mechanisms are altered during pregnancy in order to provide additional protection to the fetus and other products of conception, by preventing the ascent of micro-organisms up the reproductive tract [9]. These changes may also modulate the inflammatory processes that trigger cervical remodelling (such as cytokine-mediated synthesis of collagenases and elastases) and the uterine contractions associated with the onset of labour [9,10].

TLRs can interact with endogenous molecules released from damaged tissues or dead cells. These molecules are chronic inflammatory biomarkers or damage-associated molecular patterns (DAMPs). They regulate many sterile inflammation processes and recognize and respond to PAMPs [11,12]. DAMPs comprise High-mobility group box 1 (HMGB1), heat shock proteins (HSPs), S100 proteins, and distorted matrix proteins and play some role in initiation and progress of preterm birth (PTB) [11].

Engagement of epithelial TLRs by specific ligands leads to increased expression of mediators of inflammation, such as cytokines and chemokines, through the activation of transcriptional factors of the nuclear factor (NF)-κB family [13,14]. Increased elaboration of pro-inflammatory cytokines especially interleukin (IL)-1β, IL-6, IL-8 and TNF has been demonstrated [15]. There is emerging evidence that changes in TLR-mediated signalling during pregnancy play key roles in alterations in immune and inflammatory processes, and may be implicated in premature birth [15,16]. For instance a variant in the human TLR4 gene has been shown to be associated with an increased risk for premature birth and the secretion of pro-inflammatory cytokines [17] especially interleukin (IL)-1β, IL-6, IL-8 and TNF [18]. The release of IL-6 and IL-8 due to LPS exposure has also been shown to alter ectocervical epithelial barrier functions by increasing permeability [19,20].

We have recently observed that the expression of Toll-like receptors (TLR) -2 and -4 in human cervical tissue is increased during pregnancy [21], also reported in several other tissues during gestation [22]. However, the underlying mechanism and functional implications of these observations remain unclear. Hormones have been reported to regulate the function of several PRRs in some tissues [22,23]. We therefore hypothesised that estradiol (E_2_), an endogenous gestational hormone, may alter cervical epithelial immune-responsiveness as part of the required adaptation of reproductive tract tissue to pregnancy. In this study, we detail the effects of E_2_ on the cytokine expression profiles (as a marker of epithelial immune responsiveness) of cultured human ectocervical epithelial cells coincubated with the ligands of TLR2 (peptidoglycan, PGN) and TLR4 (lipopolysaccharide, LPS).

## 2. Materials and methods

### 2.1 Study design

The South Sheffield Research Ethics Committee (SSREC/03/105) granted approval for this study. Written informed consent from participants was obtained prior to the collection of all samples.

### 2.2 Subjects and tissue samples

Human ectocervical tissue was obtained from fresh hysterectomy specimens from 62 premenopausal patients (age range 29–50 years) undergoing their operations for benign dysfunctional uterine bleeding. All subjects had had a negative urinary pregnancy test, a normal cervical smear within the previous three years, negative swabs for genital infection, and were not taking hormonal contraceptives at least six weeks before surgery.

### 2.3 Epithelial Growth Medium (EGM)

Minimum Essential Medium (MEM) d-Valine (C-75100, Promo Cell, UK), supplemented with heat inactivated fetal bovine serum (FBS) (BioWhittaker, Lonza, Belgium Cat # DE14-820F), 0.2 mM l-glutamine, hydrocortisone (Sigma, UK Cat. # H4001) (5ml of stock solution 40 μg/ml), penicillin-streptomycin-Amphotericine B stock (Sigma, Cat # A5955), as described previously by Kamine *et*. *al*. [24], was used for culturing primary HECECs. The most important cells that can contaminate such epithelial explant cultures are fibroblasts [24–26]. Substitution of D-valine for L-valine selectively inhibits proliferation of fibroblasts, which lack the enzyme D-amino acid oxidase that converts the D-amino acid into its essential L-isoform [24,27]. MEM l-Valine without Phenol Red (Gibco, Cat. No.; 51200) and Charcoal-filtered FBS (South American origin, Cat. No. DE14-820E, Lonza) were substituted for MEM d-Valine and FBS respectively three days prior to the co-incubation experiments while hydrocortisone was omitted at this stage [28].

### 2.4 Establishment of the primary cell cultures

The tissues were collected by 8mm punch biopsies and were immediately placed in ice-cold EGM and rinsed several times with 1x phosphate buffered saline (PBS) and EGM. The epithelia were isolated carefully under the microscope, diced into 1–2 mm fragments and were then subjected to enzymatic digestion using collagenase IV (1 U/ml) (Gibco, Cat. No; 17104–019) in MEM for one hour at 37°C on a rotating surface followed by 10 minutes incubation with 1x trypsin (T3924, Sigma, UK) at 37°C [24]. The digested tissue clumps were collected and transferred to six well plates (Greiner Bio-One Ltd, Stonehouse, UK) after deactivating the trypsin. The tissue fragments were left to dry and adhere to the bottom of the wells for 10 minutes. The explants were incubated in 2 ml of EGM and maintained in a humidified incubator with 5% CO_2_ at 37°C. The medium was changed every three days. When the outgrowths of cultured HECECs reached 80% confluence (3–4 weeks), HECECs were employed for functional and gene expression studies or passaged. Cell Dissociation Solution Non-enzymatic (CDSNE) (Sigma UK, Cat. No. C5914) was used to remove the cultured cells from the culture plastic wares.

### 2.5 Fibroblast cultures

Human Neonatal Foreskin Fibroblasts (HNFF) were cultured in Dulbecco’s modified Eagle’s medium (DMEM) supplemented with 10% (v/v) fetal calf serum (FCS) and 2 mM l-glutamine. The HNFFs were kindly provided by Dr. B. Aflatoonian (Academic Unit of Reproduction and Developmental Medicine, Jessop Wing, University of Sheffield). The HNFF cultures were maintained in a humidified incubator with 5% CO_2_ at 37°C and the medium was changed every 2–3 days. The cultured HNFFs were used as positive control for CD90 staining.

### 2.6 Double immuno-fluorescence sequential staining

Primary and/or first passaged cultured HECECs on 8-well chamber slides (Falcon Fisher scientific, 08-774-25) were fixed with acetone (-20°C) for 10 min at room temperature and permeabilised by 0.1% (v/v) Triton X-100 in PBS for 10 min, followed by blocking with 3% (w/v) bovine serum albumin (BSA) for 30 minutes twice at room temperature. Primary antibodies against cytokeratin, phycoerythrin conjugated (CK PE) (ab52460, abcam, UK, Anti-pan CK 4, 5, 6, 8, 10, 13, 18) and CD90, FITC conjugated (CD90 FITC) (ab11155, abcam, UK) (specific antigens for epithelial cells and fibroblasts, respectively) were incubated with the HECECs for one hour each, at room temperature and the cells were counterstained with DAPI. Images were taken with an automated inverted microscope Leica DMI 4000B, Leica DFC300FX camera and pictures were analysed with Leica Microsystem LAS AF-AF6000 software.

### 2.7 Functional studies

#### 2.7.1 Hormonal preparation

17-β-estradiol (E_2_) (E2758, Sigma) was dissolved in absolute (100%) ethanol and MEM to the concentration of 20 μg/ml, as instructed by the manufacturer. Further dilutions were made in the MEM without Phenol Red to achieve the final working concentrations of E_2_ of 0.1 and 10 nM.

#### 2.7.2 TLR2 and TLR4 Agonists

The primary or first passaged cultured HECECs were exposed to Peptidoglycan (PGN from S. aureus, 77140, Sigma Aldrich) (50μg/ml) [29] or Lipopolysaccharide (100ng/ml) (LPS from E. coli; L-2654-1MG) [30] while being simultaneously treated with E_2_ at two different concentrations (0.1 and 10 nM) for 10 minutes, two hours and 18 hours. Three to four days prior to the stimulation with agonists of TLR2 and TLR4, the media were substituted with phenol red-free MEM (51200, Gibco) supplemented with 10% charcoal dextran-treated FBS, 0.2 mM l-glutamine and antibiotics (as mentioned in 2.3). Hydrocortisone was not added in order to avoid its estrogenic effects [29,30]. MD-2, CD14 and LPS-binding protein (LBP) were added, as they are required for optimal cellular responses to LPS [31]. These co-factors were provided with the necessary concentrations in the EGM for the LPS stimulation experiments; Recombinant Human LBP (rhLBP; 870-LP-025 R&D system) 1μg/ml [30], rhMD-2 (1787-MD R&D system) 2 ng/ml [32,33] and rhCD14 (383-CD, R&D system) 0.07 μg/ml [30].

#### 2.7.3 Evaluation of the expression of TLR2 and TLR4 by flow cytometry

Expression of TLR2 and TLR4 in the cultured HECECs were evaluated by flow cytometry using Allophycocyanin (APC) conjugated anti-human TLR2 (abcam, Cat. No. ab24996, IgG2a) and FITC conjugated anti-human TLR4 Ab (abcam, Cat. No. ab45126, IgG2b). APC conjugated rat IgG2a (eBioscience, Cat. No. 17-4724-41) and FITC conjugated rat IgG2b Isotype controls (eBioscience, Cat. No. 11-4732-41) were supplied by eBioscience. Stain Buffer (FBS, BD Pharmingen Cat. No. 554656) was provided by BD Pharmingen. The HECECs were prepared according to the suggested protocol from BD Pharmingen. Briefly, the cells were washed twice in pre-warmed PBS without Ca^2+^ & Mg^2+^ (Sigma, Cat. No. D8537) then incubated with 5ml pre-warmed CDSNE (C5914, Sigma, UK) for seven minutes at 37°C. The cells were collected in 15ml cone shaped tubes (Greiner centrifuge tubes T1818-500EA, Sigma) after detachment and 7.5ml EGM was added to each tube to deactivate CDSNE. The mixture was split into two tubes; one for gene expression study and the other for the TLRs study. The pellet was collected using 500μl of ice-cold FBS and transferred to a flow cytometry tube (ELKay Autotubes, non-sterile 1.1ml, 000-MICR-200) after centrifuging at 400 x *g* at 4°C for 5 min. Single cell suspension was prepared and washed twice using ice cold FBS and spun at 400 x *g* for 5 min. The pellet was re-suspended in 50 μl FBS and the sample was stained with 5μl APC conjugated anti-human TLR2 and 5 μl FITC conjugated anti-human TLR4 Ab. Equal volumes of APC conjugated rat IgG2a and FITC conjugated rat IgG2b Isotype controls were added to the corresponding Isotype Control sample. The samples were incubated for 30 minutes on ice protected from light. The cells were washed twice using 1 ml FBS to remove unbound antibodies. The cell pellets were re-suspended in 500 μl FBS after spinning down at 400 x *g* for 5 min and the samples were taken for cytometric evaluation within half an hour. Each set of experiments, contained 5 to 7 samples, an isotype and an unstained control each time.

### 2.8 Total RNA extraction and RT-PCR

Total RNA extraction was performed from cultured HECECs using TRI Reagent (T9424, Sigma), based on the manufacturer’s instructions. Chloroform was substituted with 1-Bromo-3-chloropropane (BCP) (B9673, Sigma) for RNA extraction to reduce the possibility of DNA contamination [34]. The eluted RNA was treated with rDNase I (Ambion^®^ DNA-*free*^™^ DNase kit, AM1906) to remove the contaminating DNA. Quantification and quality controls of the eluted RNA was carried out using the NanoDrop^TM^ 1000 Spectrophotometer (Thermo scientific) and Agilent 2100 Bioanalyser (Agilent technologies, USA) respectively. 400 ng of the eluted RNA from each sample was used for the first-strand cDNA synthesis using iScript cDNA synthesis kit (170–8890, Bio-Rad), based on the manufacturer’s instructions. RT-PCR was performed using the prepared cDNA; TLR2, TLR4, Estrogen Receptor α (ERα), ERβ, membrane Progesterone Receptor α (mPRα), mPRβ, mPRγ and nuclear progesterone receptors (nPRA & nPRB) forward and reverse primers (Table 1) and PCR Master mix, 2x (M750B, Promega Madison, USA) as described before for TLRs [35], ERs [36] and PRs [37]. All the experiments included a β-actin (positive control), a negative control with no cDNA and a No-RT control in which reverse transcriptase enzyme (RT) was excluded at the stage of cDNA synthesis. PCR products and the calibrator ladder (LowRanger DNA calibrator ladder, Cat. No. 11500, Norgen) were then resolved (10μl of each sample) through 1.2% agarose gels, and electrophoresis was run with 1x TAE buffer (Tris-acetate and EDTA) at 45V for 2.5 hours. Agarose gels were examined under a trans-illuminator and ethidium bromide (ETBr)/UV in a chemi-HR16 (LFB) G:box syngene imaging system (Syngene, UK) and digital images were taken with a GeneSnap 4.00.00 software (Synoptics Ltd).

**Table 1:** Sequence of primers for TLR2, TLR4, ERα, ERβ, mPRα, mPRβ, mPRγ, nPRA&B and β-actin.

Gene		primers 5´- 3´	Annealing Temp	Product size
TLR2	Forward	TCGGAGTTCTCCCAGTTCTCT	59.8°C	175
	Reverse	TCCAGTGCTTCAACCCACAA	57.3°C	
TLR4	Forward	CAACAAAGGTGGGAATGCTT	55.3°C	317
	Reverse	TGCCATTGAAAGCAACTCTG		
ER α	Forward	GAATCTGCCAAGGAGACTCG	59.4°C	288
	Reverse	ATCTCTCTGGCGCTTGTGTT	57.3°C	
ER β	Forward	CCAGCAATGTCACTAACTTGGA	58.4°C	217
	Reverse	TTCCCACTAACCTTCCTTTTCA	56.5°C	
mPRα	Forward	CCTGCTGTGTGATCTTAG	53.7°C	288
	Reverse	CGGAAATAGAAGCGCCA	56.0°C	
mPRβ	Forward	CACGAAGGACCCACAAAACT	57.3°C	232
	Reverse	CAATCCCAAGCACCACCTAT		
mPRγ	Forward	AGCCCCTGGACGCTTTGA	58.2°C	276
	Reverse	GGTCTGAGTCATGTTTCT	51.4°C	
nPR A&B	Forward	GCTACGAAGTCAAACCCAGT	57.3°C	274
	Reverse	CACCATCCCTGCCAATATC	56.7°C	
β-Actin	Forward	AGCATTGCTTTCGTGTAAATTATGT	56.4°C	207
	Reverse	TGGTCTCAAGTCAGTGTACAGGTAA	61.3°C	

### 2.9 Cytometric bead array

The Cytometric Bead Array technique was employed to measure eight cytokines [38] (IL-1β, IL-6, IL-8, IL-10, IL-12p70, IFNγ, RANTES and TNF) in the collected supernatant at the study time points of 10 minutes, 2hours and 18hours, to determine how E_2_ concentrations affected the responses of the cultured HECECs to stimulation with TLR2 or TLR4 ligands. Each set of experiments consisted of five samples and the baseline cytokine expression levels were determined from the supernatant fluid of HECECs stimulated with just the relevant TLR ligand. Additionally, cytokine expression profiles of non-treated HECECs were used as control. Master Buffer Kit (558264, The BD™ CBA Human Soluble Protein Flex Set System, BD Biosciences) was used for these assays and the samples were run on the BD FACS Array flow cytometry machine, using FACP Array software.

### 2.10 Statistics

Data were collected and analysed using GraphPad Prism Version 6.0f. Brown Forsythe and Bartlett’s analyses were first used to test for normality of the data. ANOVA model and Tukey's multiple comparison tests were used for the statistics. Differences were considered statistically significant at p-value less than 0.05.

## 3. Results

**Fig 1:**
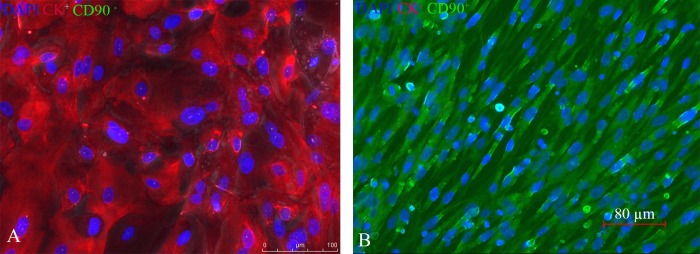
Cultured HECECs and validating their epithelial nature. (A & B) Double Immuno-Fluorescence sequential staining for HECECs and HNFF; In “A and B” both antibodies (CK and CD90) and DAPI were used. The detected red signal from the HECECs (A) demonstrates that just the Cytokeratine Ab (PE) was picked up. In “B” detected green signal represents specific Fibroblasts’ antigens identification. The nuclei have been stained with DAPI, validating the cells were alive before fixation. No green signal was detected from the cytoplasm of the culture HECECs (A); indicative of the absence of fibroblasts. HNFF; Human Neonatal Foreskin Fibroblast.

Cultures of HECECs were successfully established for all the samples (S1 Fig). The epithelial phenotype of the cultured cells was confirmed by double immunofluorescence sequential staining to detect cytokeratin (CK). The exclusion of fibroblasts from the cultures was confirmed by the absence of staining to CD90 (Fig 1A).

**Fig 2:**
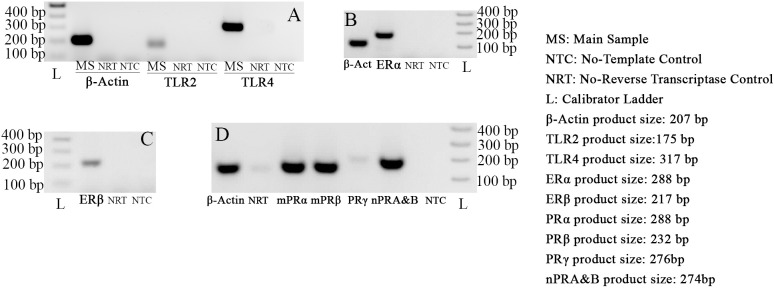
RT-PCR using RNA extracted from Cultured HECECs to investigate TLR2, TLR4, ERs and PRs gene expression. A: Detection of the signals produced by RT-PCR products for β-Actin, TLR2 and TLR4. B: Depicts detection of ERα. C: Depicts detection of ERβ. D: Signals were detected for mPRα, mPRβ, mPRγ and nPR A&B which represent the gene expression of these five receptors while the detected signals for PRγ are much weaker than the others. No signal was detected in the negative controls, representing the accuracy of the results. Presence of a faint band could be expected in the No-RT controls and it does not interfere with the accuracy of the results.

The gene expression of TLR2, TLR4, ERα, ERβ, mPRα, mPRβ, mPRγ, nPRA and nPRB were demonstrated by RT-PCR. All the amplified products were at the predicted size for the relevant genes. No signals were detected for negative control samples, indicative of absence of DNA contamination (Fig 2). The expression of ER and PR have been confirmed in previous reports [39,40].

**Fig 3:**
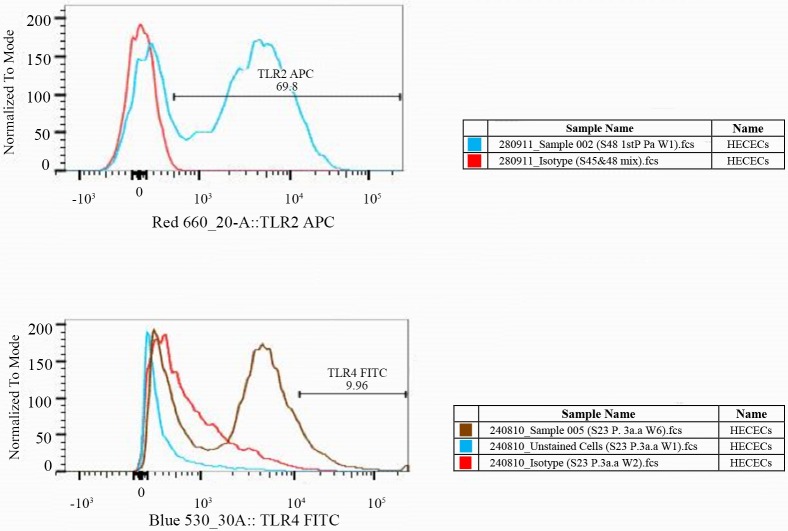
Flow cytometry results for the study of TLR2 and TLR4 expression in HECECs. A; TLR2 expression level in HECECs was revealed by detection of fluorochrome signals in histograms; Overlay histograms of isotype control (Red) and an unknown sample (Blue) stained with APC conjugated human TLR2 Ab. The fluorochrome-stained HECECs for TLR2 were highlighted within the interval gate. These defined gates were used to acquire the corresponding statistics. B; TLR4 expression level in the HECECs was revealed by detection of fluorochrome signals in histograms; Overlay histograms of isotype control (Red), unstained HECECs and an unknown sample (Brown) stained with FITC conjugated human TLR4 Ab. The fluorochrome-stained HECECs for TLR4 were highlighted within the interval gate.

The expression of TLR2 and TLR4 was successfully and consistently demonstrated in the cultured HECECs using flow cytometry (Fig 3).

### 3.1 Presence of E_2_ is associated with changes in the expression of cytokines when TLR2 and TLR4 signalling pathways are activated in cultured HECECs

#### 3.1.1 HECECs stimulated with PGN in the presence of E_2_ (Fig 4)

Estradiol did not have a consistent effect on basal release of most cytokines studied: compared to control, expression levels of IL-1ß, IL-6, IL-10, IFNγ and RANTES were largely unchanged but the basal expression levels of IL-8 were suppressed. PGN stimulated the secretion of IL-1ß, IL-6, IL-8, IFNγ, and RANTES, mostly after 18h of co-incubation. However, PGN did not appear to stimulate IL-10 secretion by HECECS. With PGN stimulation the HECECs exposed to E_2_ released significantly less IL-1β, IL-6 (18h) and IFNγ, and significantly more IL-8 (10min and 2h), IL-6 (10min and 2h) and RANTES (10min, 2h and 18h), than non-E_2_ treated controls.

**Fig 4:**
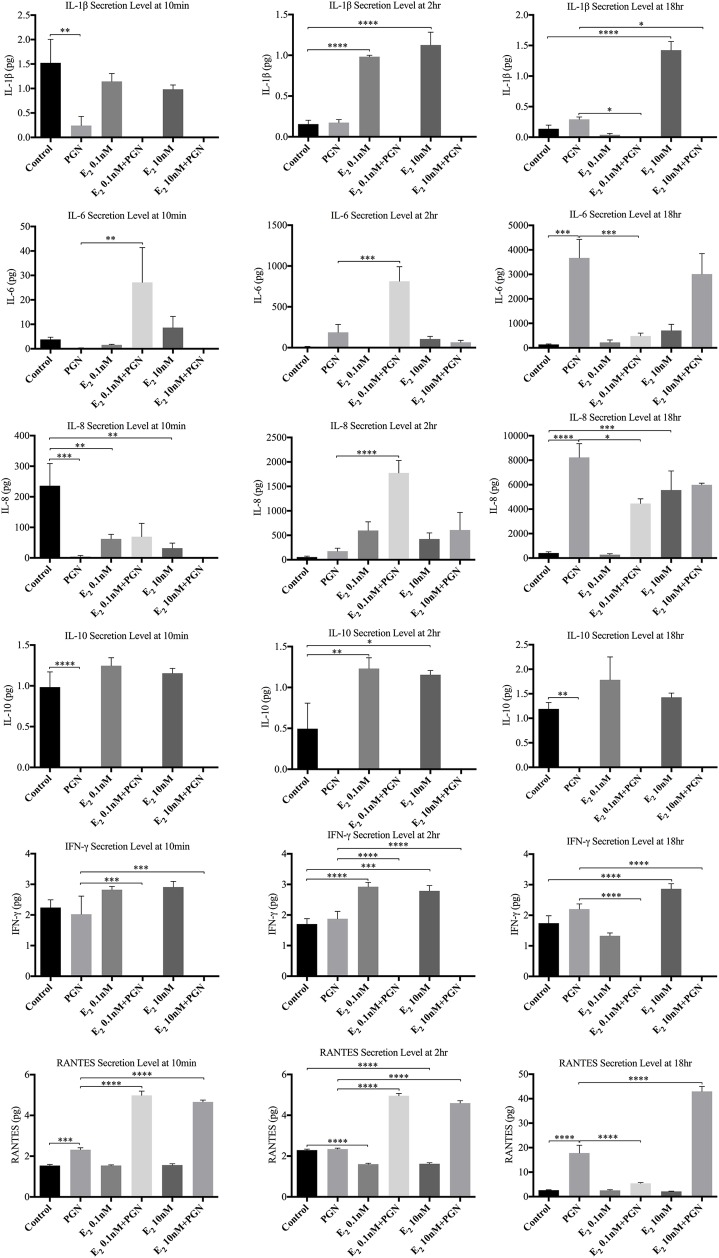
Detected cytokine secretion profiles when the cultured HECECs were stimulated with TLR2 agonist. Demonstrates significant changes in five out of eight studied cytokines when the HECECs were stimulated with PGN in the presence of two different concentrations of E_2_ compared to non-E_2_-treated. *p value <0.05, **p value<0.005, ***p value<0.0005, ****p value<0.0001.

#### 3.1.2 HECECs stimulated with LPS in the presence of E_2_ (Fig 5)

During these experiments estradiol did not have a consistent effect on basal release of most cytokines studied: compared to control, expression levels of all cytokines by untreated HECECs varied markedly at all time points studied, with RANTES being consistently suppressed or unchanged. LPS stimulated the sustained secretion of IL-6, IL-8, and RANTES by untreated HECECS and had minimal or no effects on the expression levels of the other cytokines studied. Compared to non-E_2_ treated controls, LPS stimulation of HECECs treated with E_2_ induced increased amounts of IL-1β after 18h. Decreased secretion of RANTES was observed with E_2_ treatment after 10 min, 2 and 18h. Conversely, IFNγ was decreased after 10min followed by enhanced expression levels at 18h.

**Fig 5:**
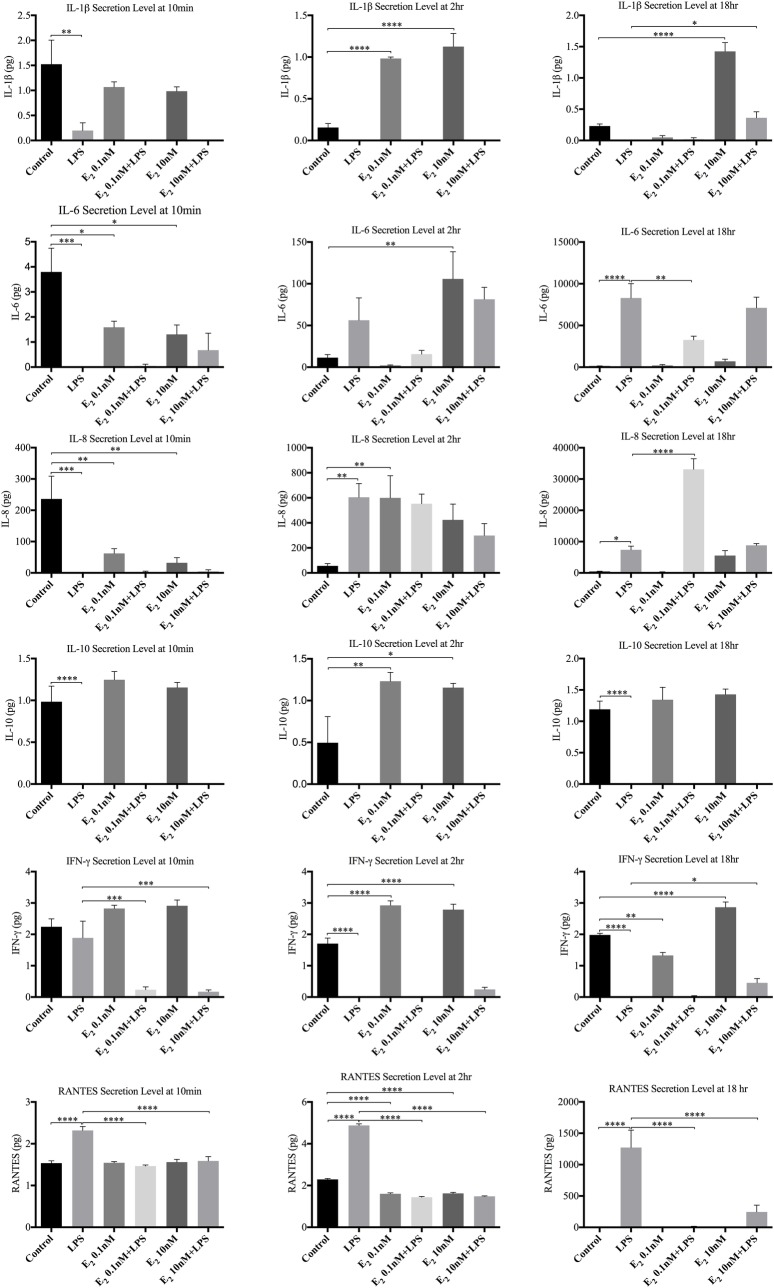
Detected cytokine secretion profiles when the cultured HECECs were stimulated with TLR4 agonist. Depicts significant alteration in five out of eight studied cytokine levels when the HECECs were stimulated with LPS in the presence of two different E_2_ concentrations compared to non-E_2_-treated HECECs. *p value <0.05, **p value<0.005, ***p value<0.0005, ****p value<0.0001.

## 4. Discussion

We have demonstrated that E_2_ alters the cytokine responses of cultured HECECs when TLR2 and TLR4 signalling pathways are activated. However, the nature of the altered response varies by cytokine and the duration of coincubation of HECECs with E_2_ and TLRs ligand. Whilst E_2_ appears to reduce the release of three of the eight cytokines measured (IL-1β, IL-6, IFNγ) when TLR2 is stimulated by PGN, it enhances the release of the same cytokines as well as IL-8 when the TLR4 receptor is activated by LPS. Whilst the TLR2 ligand, PGN induces enhanced expression levels of RANTES in the presence of E_2_, LPS suppresses RANTES from E_2_-exposed HECECs. Our observations suggest that E_2_ modulates the immune responsiveness of cultured primary HECECs exposed to exogenous ligands of TLR2 and TLR4 in a complex and varied fashion.

In order to assess the immune-responsiveness of HECECs, we have studied their cytokine expression response to ligand stimulation of the TLR2 and TLR4 pattern recognition receptors, which respond to gram-positive and gram-negative bacteria, respectively. Some of these pathogens cause sepsis of the female reproductive tract [41]. Inflammatory responses may be promoted by stimulation of either endogenous or exogenous ligands of TLR2 and or TLR4. We aimed to determine whether E_2_, a hormone that crucially drives the changes in the female reproductive tract during the menstrual cycle as well as during pregnancy, alters the immune-responsiveness of cervical epithelium. It has been observed that E_2_ can attenuate inflammation at different physiological or supra-physiological levels, appearing to generally promote pro-inflammatory pathways at lower physiologic levels [42]. Having established that PGN and LPS increased expression levels of different cytokines by HECECs, we demonstrated that these responses were significantly altered by co-incubation of HECECs with E_2_. The observed effects varied with the duration of cell exposure to E_2_, suggesting that the effects observed after 10 min of E_2_ exposure could have a different underlying signalling mechanism to those observed after 2 or 18h.

It has been recognised that there are multifaceted mechanisms involved with estrogen receptor (ER) biological signalling [43]. More than four ER pathways have been described. These include: a) the classical ligand-dependent pathway that involves E_2_-ER complexes binding to estrogen response elements (ERE) in target promoters leading to an up- or down-regulation of gene transcription and subsequent tissue responses; b) the ligand-independent pathways through which growth factors (GF) or cyclic AMP activate intracellular kinase pathways, leading to phosphorylation and activation of ER in a ligand-independent manner; and c) the ERE-independent signalling that involves E_2_-ER complexes altering transcription of genes through association with other DNA-bound transcription factors that tether the activated ER to DNA, resulting in an up-regulation of gene expression. Finally, it has long been recognised that cell-surface (non-genomic) E_2_ signalling occurs by the activation of a putative membrane-associated binding site, possibly a form of ER linked to intracellular signal transduction pathways that generate rapid tissue responses. It is unclear which of these pathways is involved in our observations, but it is highly likely that the effects of E_2_ on TLR2- and TLR4-mediated ligand activity in HECECs noted after 10 min are membrane-associated non-genomic effects [44,45], whilst other nuclear genomic pathways are likely to mediate the sustained cytokine responses noted after 2 to 18 h. Interestingly, the effects of E_2_ on LPS-induced cytokine expression profiles by HECECs are remarkably similar at 2 and 18 h but often dissimilar or opposite to observations after 10 min during the same experiments.

The binding of E_2_ to the ER initiates the relevant ligand-dependent E_2_ signalling. The subsequent cell-specific transcriptional response to E_2_ is determined by multiple factors; the composition of co-regulatory proteins in a given cell and the characteristics of the promoters of estrogen responsive genes are the most important factors. Since hormones are modulators of transcription, the pattern of modulated genes also depends on what other signalling pathways are active in the cell at the time of hormone exposure [46,47]. This may explain how E_2_ can differentially modify immune responses of HECECs when either TLR2 or TLR4 downstream signalling pathways is activated.

Substantial signalling complexity, consistent with our observations, is suggested by reports that individual cytokine responses to ligand-mediated immune cell activation can vary greatly with tissue and duration of stimulation [48]. Such complexity has led to the investigation of cytokine profiles in various disease and physiological states in an attempt to define cytokine “signatures” that facilitate disease diagnosis, assessment of associated morbidity, and for monitoring therapy. Distinctive disease-specific cytokine profiles have been identified in inflammatory bowel disease and have been demonstrated to show significant correlations to disease activity and duration [49]. Whether the changes in cytokine expression that we have described reflect consistent E_2_-induced gestational changes required to combat infection and modulate inflammation remains to be investigated. It is plausible that PAMPs, DAMPs and gestational hormones may drive the changes in cytokine expression profiles by cervical epithelial cells required to combat sepsis or influence cervical remodelling preparatory to birth [11]. These in vivo gestational changes in immune-responsiveness of cervical epithelial cells are likely to be more complex than we have observed in our experiments because several other hormones (such as progesterone) and tissue factors are likely to play significant roles.

Our observations suggest that cytokine expression profiles are likely to be altered during human pregnancy under hormonal influence, compared to the nonpregnant state. However, the nature of such change and its physiological relevance remains to be determined. Studies have highlighted the potential pathogenic role for these changes in the aetiology of preterm birth. One study noted that a higher proportion of women with low levels of IL-1α (below 25^th^ percentile) delivered preterm compared to women with higher IL-1α levels, suggesting that the rate of preterm delivery increased when IL-1α levels decreased [50]. Another study also demonstrated that women in the lowest quartile of cervical concentrations of IL-1β and IL-8 early in pregnancy were significantly more likely to subsequently experience chorioamnionitis than women in higher quartiles. We observed sustained increased expression of IL-1ß, IL-8 and IFNγ by HECECs stimulated with LPS in the presence of E_2_: this could represent an immunoprotective effect conferred on these cells by E_2_, thus providing protection of the conceptus against ascending infection and perhaps preventing inflammation-induced preterm birth. In contrast, we observed that E_2_ appears to predominantly reduce pro-inflammatory cytokine expression of HECECs to PGN, TLR2 ligand. The reason for this observation is unclear. It may reflect an anti-inflammatory response of HECECs to those bacterial ligands that would otherwise induce inappropriate inflammation in the lower reproductive tract. Whether this process may be accentuated during pregnancy to confer additional protection against infection to the conceptus remains to be determined. Estradiol also appears to suppress the release of RANTES, a chemotactic cytokine, by HECECs exposed to LPS whilst increasing RANTES release by HECECs exposed to PGN, suggesting a ligand-specific modulatory effect of estradiol on HECECs.

There is a paucity of studies investigating commensal-host immunologic interactions in the female reproductive tracts. However insight into TLR-commensal interactions is gleaned from studies of the gut where it has been demonstrated that the microbial ligands recognized by TLRs are not unique to pathogens, being also produced by commensal microorganisms [51]. These studies have demonstrated several mechanisms by which intestinal epithelial immunological homeostasis is maintained despite exposure to commensals and pathogens. Firstly, surface epithelium may sequester bacteria on contact thereby avoiding mounting an inflammatory response. Secondly, commensal microflora may activate TLRs in a way that prevents tissue injury and associated mortality [51]. One such mechanism could be the paradoxic reduction in cytokine production by epithelial cells when exposed to PAMPs from commensals rather than pathogens. This may explain our observations in regard to PGN. Overall, the varied cytokine responses of E_2_-treated cervical epithelial tissue to TLR2 and TLR4 ligands, suggest complex host-microbial interactions required by tissues that are exposed to both commensal and pathogenic florae.

In addition to their role in host defence against infections, these cytokines may also modulate cervical remodelling of sub-epithelial cervical matrix during pregnancy, perhaps by inducing chemokines and local tissue infiltration by white cells. They may also cause vascular changes that induce matrix breakdown and alter tissue hydration [11]. The pattern of cytokine expression under the influence of E_2_, may change the polarisation of the immune responses from a Th2 to a Th1 type. Activation of Th1-type immune responses can initiate the final rapid stage of softening of the uterine cervix by inducing neutrophil infiltration and increasing the activity of proteases such as collagenase and elastase, and prostaglandins [11]

Our *in vitro* studies of E_2_ on isolated cervical epithelial cells have several limitations. Whilst it enables characterisation of the modulatory role of E_2_ on the immune-responsiveness of these cells, it does not take into account the associated, rather more complex, influence of other gestational hormones such as progesterone on the immune-responsiveness of this reproductive tract epithelium [52,53]. Furthermore the precise role of endogenous ligands of TLRs and DAMPs, believed to play key role(s) in receptor activation and the pathogenesis of preterm birth [11,12] require further studies. It is likely that E_2_ and the innate immune system maintain an active interaction during normal pregnancy even without exogenous PAMPs. Understanding the mediators of such interaction would shed further light on the observed difference between the effects of TLR2 and TLR4 ligands on cytokines in the presence of E2, as well as the mechanisms of cervical tissue remodeling associated with premature birth.

In conclusion, HECECs cultured in the presence of E_2_ demonstrate altered cytokine expression profiles in response to TLR2 and TLR4 ligands. The varied nature and time course of these changes suggest a complex immune-modulatory role for E_2_ at this epithelial surface. Such a role would enable the mucosa of the lower reproductive tract discriminate between commensals and pathogens, and mount appropriate host defence against ascending infection in the pregnant and non-pregnant state. The signalling mechanisms for these observations remain to be elucidated.

## Supporting information

S1 Fig Culturing HECECs from digested diced tissue. (A&B); Pieces of tissue (Ectocervix) with growing HECECs have been shown. “e”; Explant, “g”; Growing HECECs.
(TIF)
