# Supplementary figures and images for: Estradiol alters the immune-responsiveness of cervical epithelial cells stimulated with ligands of Toll-like receptors 2 and 4
Source: PLoS One. 2017 Mar 15;12(3):e0173646. doi: 10.1371/journal.pone.0173646 (PMC5351915; doi:10.1371/journal.pone.0173646)

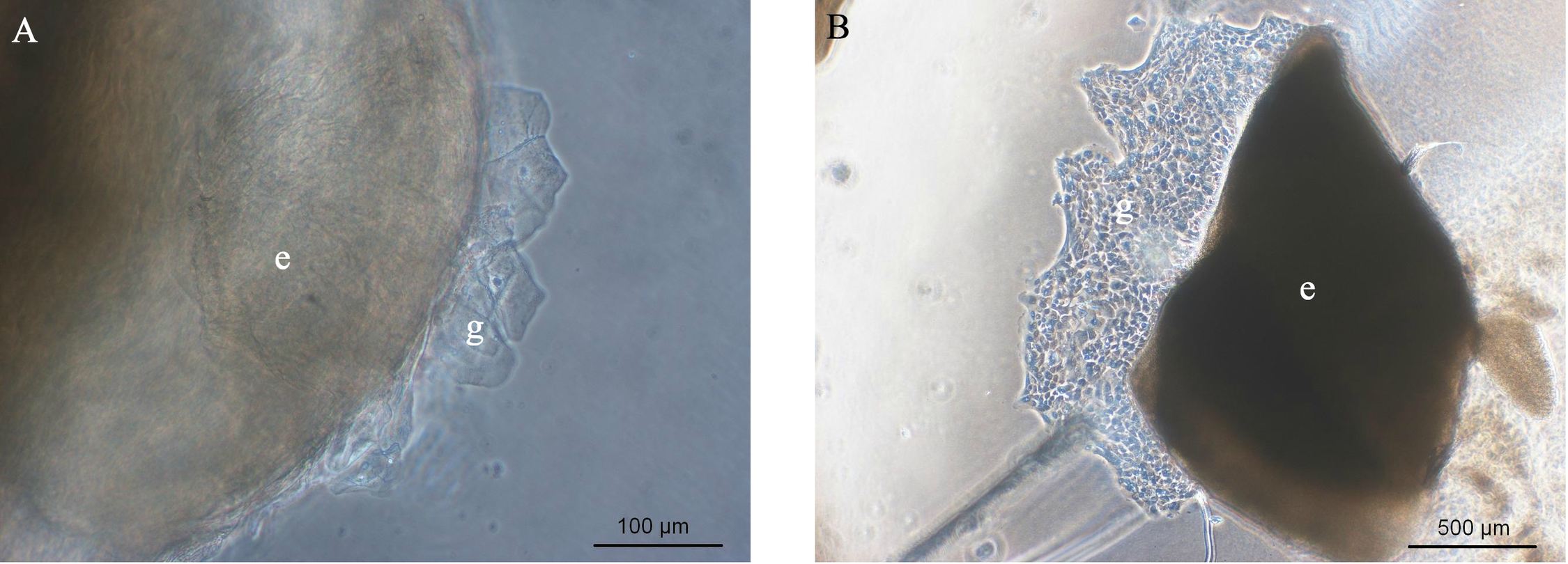

Supplement: S1 Fig — (A&B); Pieces of tissue (Ectocervix) with growing HECECs have been shown. “e”; Explant, “g”; Growing HECECs. (TIF) [file pone.0173646.s001.tif]
